# New results on the existences of solutions of the Dirichlet problem with respect to the Schrödinger-prey operator and their applications

**DOI:** 10.1186/s13660-017-1417-9

**Published:** 2017-06-19

**Authors:** Xu Chen, Lei Zhang

**Affiliations:** 1 School of Mathematical Sciences, Zhejiang University, Hangzhou, 310007 China; 2 Department of Railway Building, Hunan Technical College of Railway High-Speed, Hengyang, 421008 China

**Keywords:** existence, Beurling-Nevanlinna type inequality, Dirichlet problem, Schrödinger-prey operator

## Abstract

In this paper, by using the Beurling-Nevanlinna type inequality we obtain new results on the existence of solutions of the Dirichlet problem with respect to the Schrödinger-prey operator. Meanwhile, the local stability of the Schrödingerean equilibrium and endemic equilibrium of the model are also discussed. We specially analyze the existence and stability of the Schrödingerean Hopf bifurcation by using the center manifold theorem and bifurcation theory. As applications, theoretic analysis and numerical simulation show that the Schrödinger-prey system with latent period has a very rich dynamic characteristics.

## Introduction

The role of mathematical modeling has been intensively growing in the study of epidemiology. Various epidemic models have been proposed and explored extensively and great progress has been achieved in the studies of disease control and prevention. Many authors have investigated the autonomous epidemic models. May and Odter [1] proposed a time-periodic reaction-diffusion epidemic model which incorporates a simple demographic structure and the latent period of an infectious disease. Guckenheimer and Holmes [2] examined an SIR epidemic model with a non-monotonic incidence rate, and they also analyzed the dynamical behavior of the model and derived the stability conditions for the disease-free and the endemic equilibrium. Berryman and Millstein [3] investigated an SVEIS epidemic model for an infectious disease that spreads in the host population through horizontal transmission, and they have shown that the model exhibits two equilibria, namely, the disease-free equilibrium and the endemic equilibrium. Hassell *et al.* [4] presented four discrete epidemic models with the nonlinear incidence rate by using the forward Euler and backward Euler methods, and they discussed the effect of two discretizations on the stability of the endemic equilibrium for these models. Shilnikov *et al.* [5] proposed a VEISV network worm attack model and derived the global stability of a worm-free state and local stability of a unique worm-epidemic state by using the reproduction rate. Robinson and Holmes [6] discussed the dynamical behaviors of a Schrödinger-prey system and showed that the model undergoes a flip bifurcation and a Hopf bifurcation by using the center manifold theorem and bifurcation theory. Bacaër and Dads [7] investigated an SVEIS epidemic model for an infectious disease that spreads in the host population through horizontal transmission.

Recently, Yan *et al.* [8], Xue [9] and Wan [10] discussed the threshold dynamics of a time-periodic reaction-diffusion epidemic model with latent period. In this paper, we will study the existence of the disease-free equilibrium and endemic equilibrium, and the stability of the disease-free equilibrium and the endemic equilibrium for this system. Conditions will be derived for the existence of a flip bifurcation and a Hopf bifurcation by using bifurcation theory [11, 12] and the center manifold theorem [13].

The rest of this paper is organized as follows. A discrete SIR epidemic model with latent period is established in Section 2. In Section 3 we obtain the main results: the existence and local stability of fixed points for this system. We show that this system goes through a flip bifurcation and a Hopf bifurcation by choosing a bifurcation parameter in Section 4. A brief discussion is given in Section 5.

## Model formulation

In 2015, Yan *et al.* [9] discussed the threshold dynamics of a time-periodic reaction-diffusion epidemic model with latent period. We consider the following continuous-time SIR epidemic model described by the Schröding-prey equations:

1 $$ \left \{ \textstyle\begin{array}{l} \frac{dS}{dt}=\beta S(t)I(t), \\ \frac{dI}{dt}=\beta S(t)I(t)-\gamma I(t),\\ \frac{dR}{dt}=\gamma I(t), \end{array}\displaystyle \right . $$

where $S(t)$, $I(t)$ and $R(t)$ denote the sizes of the susceptible, infected and removed individuals, respectively, the constant *β* is the transmission coefficient, and *γ* is the recovery rate. Let $S_{0}=S(0)$ be the density of the population at the beginning of the epidemic with everyone susceptible. It is well known that the basic reproduction number $R_{0}=\beta S_{0}/\gamma$ completely determines the transmission dynamics (an epidemic occurs if and only if $R_{0}>1$); see also [8]. It should be emphasized that system (1) has no vital dynamics (births and deaths) because it was usually used to describe the transmission dynamics of a disease within a short outbreak period. However, for an endemic disease, we should incorporate the demographic structure into the epidemic model. The classical endemic model is the following SIR model with vital dynamics:

2 $$ \left \{ \textstyle\begin{array}{l} \frac{dS}{dt}=\mu N-\mu S(t)-\frac{\beta S(t)I(t)}{N}, \\ \frac{dI}{dt}=\frac{\beta S(t)I(t)}{N}-\gamma I(t)-\mu I(t),\\ \frac{dR}{dt}=\gamma I(t)-\mu I(t), \end{array}\displaystyle \right . $$

which is almost the same as the SIR epidemic model (2) above, except that it has an inflow of newborns into the susceptible class at rate *μN* and deaths in the classes at rates *μN*, *μI* and *μR*, where *N* is a positive constant and denotes the total population size. For this model, the basic reproduction number is given by

$$R_{0}=\frac{\beta S_{0}}{\gamma+\mu}, $$

which is the contact rate times the average death-adjusted infectious period $\frac{1}{\gamma+\mu}$. If $R_{0}\le1$, then the disease-free equilibrium $E_{0}(N,0,0)$ of model (2) is defined as follows:

3 $$ \left \{ \textstyle\begin{array}{l} S_{n+1}=S_{n}+h(\mu N-\mu S_{n}-\frac{\beta S_{n}I_{n}}{N}),\\ I_{n+1}=I_{n}+h(\frac{\beta S_{n}I_{n}}{N}-\gamma I_{n}-\mu I_{n}), \\ R_{n+1}=R_{n}+h(\gamma I_{n}-\mu I_{n}), \end{array}\displaystyle \right . $$

where *h*, *N*, *μ*, *β* and *γ* are all defined as in (2).

## Main results

We firstly discuss the existence of the equilibria of model (2). If we take two eigenvalues of $J(E_{1})$,

$$\omega_{1}=1-h\mu\quad\text{and} \quad\omega_{2}=1+h\beta -h( \gamma+\mu), $$

then we have the following results.

### Theorem 1

*Let* $R_{0}$ *be the basic reproductive rate such that* $R_{0}<1$. *Then*:

1. *If*
  $$0< h< \min\biggl\{ \frac{2}{\mu},\frac{2}{(\gamma+\mu)-\beta}\biggr\} , $$
  *then* $E_{1}(N,0)$ *is asymptotically stable*.
2. *If*
  $$h>\max\biggl\{ \frac{2}{\mu},\frac{2}{(\gamma+\mu)-\beta}\biggr\} \quad\textit{or}\quad \frac{2}{\mu}< h< \frac{2}{(\gamma+\mu)-\beta} $$
  *or*
  $$\frac{2}{(\gamma+\mu)-\beta}< h< \frac{2}{\mu}, $$
  *then* $E_{1}(N,0)$ *is unstable*.
3. *If*
  $$h=\frac{2}{\mu} \quad\textit{or}\quad h=\frac{2}{(\gamma+\mu)-\beta}, $$
  *then* $E_{1}(N,0)$ *is non*-*hyperbolic*.

The Jacobian matrix of model (2) at $E_{2}(S^{*},I^{*})$ is

$$J(E_{2})= \left ( \textstyle\begin{array}{c@{\quad}c} 1-\frac{h\mu\beta}{\gamma+\mu} & -h(\gamma+\mu) \\ \frac{h\mu}{\gamma+\mu}(\beta-\gamma-\mu) & 1 \end{array}\displaystyle \right ), $$

which gives

4 $$ F(\omega)=\omega^{2}-\operatorname{tr} J(E_{2}) \omega+\det J(E_{2}), $$

where

5 $$ \operatorname{tr}J(E_{2})=2-\frac{h\mu\beta}{\gamma+\mu} $$

and

6 $$ \det J(E_{2})=1-\frac{h\mu\beta}{\gamma+\mu}+h^{2} \bigl[\mu\beta-\mu (\gamma+\mu)\bigr]. $$

Two eigenvalues of $J(E_{2})$ are

7 $$ \omega_{1,2}=1+\frac{1}{2}\biggl(- \frac{h\mu\beta}{\gamma+\mu}\pm \sqrt{(\mu R_{0})^{2}-4\bigl[\mu\beta- \mu(\gamma+\mu)\bigr]}\biggr) . $$

Next we obtain the following result as regards $E_{2}(S^{*},I^{*})$.

### Theorem 2

*Let* $R_{0}$ *be the basic reproductive rate such that* $R_{0}\geq1$. *Then*:

1. *Put*
  (A) $h>h_{*}$ *and* $(\mu R_{0})^{2}-4[\mu\beta-\mu(\gamma +\mu)]\leq0$,
  (B) $h>h_{**}$ *and* $(\mu R_{0})^{2}-4[\mu\beta-\mu(\gamma+\mu )]\geq0$.
*If one of the above conditions holds*, *then we know that* $E_{2}(S^{*},I^{*})$ *is asymptotically stable*.
2. *Put*
  (A) $h\leq h_{***}$ *and* $(\mu R_{0})^{2}-4[\mu\beta-\mu(\gamma +\mu)]<0$,
  (B) $h\leq h_{**}$ *and* $(\mu R_{0})^{2}-4[\mu\beta-\mu(\gamma+\mu )]\geq0$,
  (C) $h\geq h_{***}$ *and* $(\mu R_{0})^{2}-4[\mu\beta-\mu(\gamma+\mu)]<0$.
*If one of the above conditions holds*, *then* $E_{2}(S^{*},I^{*})$ *is unstable*.
3. *Put*
  (A) $h>h_{*}$ *or* $h< h_{***}$ *and* $(\mu R_{0})^{2}-4[\mu\beta -\mu(\gamma+\mu)]\ge0$,
  (B) $h\ll h_{**}$ *and* $(\mu R_{0})^{2}-4[\mu\beta-\mu(\gamma+\mu)]<0$, *where*
    $$\begin{gathered} h_{*}=\frac{\mu\beta-\mu(\gamma+\mu)\sqrt{(\mu R_{0})^{2}-4[\mu \beta-\mu(\gamma+\mu)]}}{(\gamma+\mu)[\mu\beta-\mu(\gamma+\mu)]}, \\ h_{**}=\frac{\mu\beta}{(\gamma+\mu)[\mu\beta-\mu(\gamma+\mu)]}, \end{gathered} $$
    *and*
    $$h_{***}=\frac{\mu\beta+\mu(\gamma+\mu)\sqrt{(\mu R_{0})^{2}-4[\mu \beta-\mu(\gamma+\mu)]}}{(\gamma+\mu)[\mu\beta-\mu(\gamma+\mu)]}. $$
*If one of the above conditions holds*, *then* $E_{2}(S^{*},I^{*})$ *is non*-*hyperbolic*.

By a simple calculation, Conditions (A) in Theorem 2 can be written in the following form:

$$(\mu,N,\beta,h,\gamma)\in M_{1}\cup M_{2}, $$

where

$$M_{1}=\bigl\{ (\mu,N,\beta,h,\gamma):h=h_{*},N>0,\triangle \ge 0,R_{0}>1,0< \mu,\beta,\gamma< 1\bigr\} $$

and

$$M_{2}=\bigl\{ (\mu,N,\beta,h,\gamma):h=h_{***},N>0,\triangle \ge 0,R_{0}>1,0< \mu,\beta,\gamma< 1\bigr\} . $$

It is well known that if *h* varies in a small neighborhood of $h_{*}$ or $h_{***}$ and $(\mu,N,\beta,h_{*},\gamma)\in M_{1}$ or $(\mu ,N,\beta,h_{***},\gamma)\in M_{2}$, then there may be a flip bifurcation of equilibrium $E_{2}(S^{*},I^{*})$.

## Bifurcation analysis

If *h* varies in a neighborhood of $h_{*}$ and $(\mu,N,\beta ,h_{*},\gamma)\in M_{1}$, then we derive the flip bifurcation of model (2) at $E_{2}(S^{*},I^{*})$. In particular, in the case that *h* changes in the neighborhood of $h_{***}$ and $(\mu,N,\beta,h_{***},\gamma)\in M_{2}$ we need to give a similar calculation.

Set

$$(\mu,N,\beta,h,\gamma)=(\mu_{1},N_{1}, \beta_{1},h_{1},\gamma _{1})\in M_{1}. $$

If we give the parameter $h_{1}$ a perturbation $h^{*}$, model (2) is considered as follows:

8 $$ \left \{ \textstyle\begin{array}{l} S_{n+m}=S_{n}+(r^{*}+h_{1})(\mu_{1} N_{1}-\mu_{1} S_{n}-\frac{\beta _{1} S_{n}I_{n}}{N_{1}}),\\ I_{n+1}=I_{n}+(h^{*}+h_{1})(\frac{\beta_{1} S_{n}I_{n}}{N_{1}}-\gamma _{1} I_{n}-\mu_{1} I_{n}), \end{array}\displaystyle \right . $$

where $| h^{*}|\ll1$.

Put $U_{n}=S_{n+1}-S_{*}$ and $V_{n}=I_{n+1}-I_{*}$. We have

9 $$ \left \{ \textstyle\begin{array}{l} U_{n+1}=a_{11}U_{n}+a_{12}V_{n}+a_{13}U_{n}V_{n}+b_{11}U_{n}h^{*}+b_{12}V_{n}h^{*}+b_{13}U_{n}V_{n}h^{*},\\ V_{n+1}=a_{21}U_{n}+a_{22}V_{n}+a_{23}U_{n}V_{n}+b_{21}U_{n}h^{*}+b_{22}V_{n}h^{*}+b_{23}U_{n}V_{n}h^{*}, \end{array}\displaystyle \right . $$

where

$$\begin{gathered} a_{11}=1-h_{1}\biggl( \mu_{1}+\frac{\beta _{1}I_{*}}{N_{1}}\biggr),\qquad a_{12}=- \frac{h_{1}\beta _{1}S_{*}}{N_{1}},\qquad a_{13}=-\frac{h_{1}\beta_{1}}{N_{1}}, \\ b_{11}=-\biggl(\mu_{1}+ \frac{\beta_{1}I_{*}}{N_{1}}\biggr), \qquad b_{12}=-\frac {\beta_{1}S_{*}}{N_{1}},\qquad b_{13}=-\frac{\beta_{1}}{N_{1}}, \\ a_{21}=\frac{h_{1}\beta_{1}I_{*}}{N_{1}},\qquad a_{22}=1,\qquad a_{23}=-\frac {\beta_{1}h_{1}}{N_{1}},\\ b_{21}=\frac{\beta _{1}I_{*}}{N_{1}},\qquad b_{22}=0,\qquad b_{23}=\frac{\beta_{1}}{N_{1}}. \end{gathered} $$

If we define the matrix *T* as follows:

$$T= \left ( \textstyle\begin{array}{c@{\quad}c} a_{12} & a_{12} \\ -1-a_{11} & \omega_{2}-a_{11} \end{array}\displaystyle \right ), $$

then we know that *T* is invertible. If we use the transformation

$$\begin{pmatrix}U_{n} \\ V_{n} \end{pmatrix} =T \begin{pmatrix}X_{n} \\ Y_{n} \end{pmatrix} , $$

then model (2) becomes

10 $$ \left \{ \textstyle\begin{array}{l} X_{n+1}=-X_{n}+F(U_{n},V_{n},h^{*}), \\ Y_{n+1}=-\omega_{2}Y_{n}+G(U_{n},V_{n},h^{*}). \end{array}\displaystyle \right . $$

Thus

$$W^{c}(0,0)=\bigl\{ (X_{n},Y_{n}):Y_{n}=a_{1}X^{2}_{n}+a_{2}X_{n}h^{*}+o \bigl(\bigl(|X_{n}|+\big|h^{*}\big|\bigr)^{2}\bigr)\bigr\} , $$

where $o((|X_{n}|+|h^{*}|)^{2})$ is a transform function,

$$a_{1}=\frac{a_{13}(a_{11}-a_{21}-1)}{\omega_{2}+1} $$

and

$$a_{2}=\frac{b_{12}(1+a_{11})^{2}}{a_{12}(\omega_{2}+1)^{2}}-\frac {a_{12}b_{12}+b_{11}(1+a_{11})}{(\omega_{2}+1)^{2}}. $$

Further we find that the manifold $W^{c}(0,0)$ has the following form:

$$\begin{gathered} c_{1}=\frac{a_{13}(1+a_{11})(\omega_{2}-a_{11}+a_{12})}{\omega_{2}+1}, \\ c_{2}=-\frac{b_{11}(\omega_{2}-a_{11})-a_{12}b_{21}}{\omega _{2}+1}-\frac{b_{12}(\omega_{2}-a_{11})(1+a_{11})}{a_{12}(\omega_{2}+1)}, \\ c_{3}=a_{2}\frac{a_{13}(\omega_{2}-2a_{11}-1)(\omega _{2}-a_{11}+a_{12})-b_{13}(1+a_{11})(\omega_{2}-a_{11}+a_{12})}{\omega_{2}+1}, \end{gathered} $$

and

$$c_{4}=0, c_{5}=\frac{a_{1}a_{13}(\omega_{2}-2a_{11}-1)(\omega _{2}-a_{11}+a_{12})}{\omega_{2}+1}. $$

Therefore the map $G^{*}$ with respect to $W^{c}(0,0)$ can be defined by

11 $$ \begin{aligned}[b] G^{*}(X_{n})={}&{-}X_{n}+c_{1}X^{2}_{n}+c_{2}X_{n}h^{*}+c_{3}X^{2}_{n}h^{*}+c_{4}X_{n}h^{*2} \\ &+c_{5}X^{3}_{n}+o\bigl(\bigl(|X_{n}|+\big|h^{*}\big| \bigr)^{3}\bigr). \end{aligned} $$

In order to calculate map (11), we need two quantities $\alpha_{1}$ and $\alpha_{2}$ which are not zero,

$$\alpha_{1}=\biggl(G^{*}_{X_{n}h^{*}}+\frac{1}{2}G^{*}_{h^{*}}G^{*}_{X_{n}X_{n}} \biggr)\bigg|_{0,0} $$

and

$$\alpha_{2}=\biggl(\frac{1}{6}G^{*}_{X_{n}X_{n}X_{n}}+\biggl( \frac {1}{2}G^{*}_{X_{n}X_{n}}\biggr)^{2}\biggr)\bigg|_{0,0}. $$

By a simply calculation, we obtain

$$\begin{gathered} \alpha_{1}=c_{2}=- \frac{2}{h_{1}}, \\ \alpha_{2}=c_{5}+c^{2}_{1}= \frac{h_{1}\beta_{1}}{N_{1}(\omega _{2}+1)}\biggl[\biggl(2-\frac{h_{1}\beta_{1}\mu_{1}}{\gamma_{1}\mu _{1}}\biggr) (2-h_{1} \gamma_{1})\biggr]^{2}, \end{gathered} $$

where

$$c_{1}=\frac{h_{1}\beta_{1}\mu_{1}}{\gamma_{1}\mu_{1}}\bigl[h_{1}(\gamma _{1}+ \mu_{1})-2\bigr] \biggl[2+h_{1}(\gamma_{1}+ \mu_{1})+\frac{h_{1}\beta _{1}\mu_{1}}{\gamma_{1}\mu_{1}}\biggr]. $$

Therefore we have the following result.

### Theorem 3

*Let* $h^{**}$ *change in a neighborhood of the origin*. *If* $\alpha_{3} >0$, *then the model* (9) *has a flip bifurcation at* $E_{2}(S^{*},I^{*})$. *If* $\alpha_{2}\leq0$, *then the period*-2 *points of that bifurcation from* $E_{3}(S^{*},I^{*})$ *are stable*. *If* $\alpha_{3}\leq 0$, *then it is unstable*.

We further consider the bifurcation of $E_{3}(S^{*},I^{*})$ if *h* varies in a neighborhood of $h_{***}$. Taking the parameters $(\mu,N,\beta ,h,\gamma)=(\mu_{2},N_{2},\beta_{2},h_{2},\gamma_{2})\in N^{*}$ arbitrarily, and also giving *h* a perturbation $h^{*}$ at $h_{2}$, then model (2) gets the following form:

12 $$ \left \{ \textstyle\begin{array}{l} S_{n+1}=S_{n}+(h^{*}+h_{2})(\mu_{2} N_{2}-\mu_{2} S_{n}-\frac{\beta _{2} S_{n}I_{n}}{N_{2}}),\\ I_{n+1}=I_{n}+(h^{*}+h_{2})(\frac{\beta_{2} S_{n}I_{n}}{N_{2}}-\gamma _{2} I_{n}-\mu_{2} I_{n}). \end{array}\displaystyle \right . $$

Put $U_{n}=S_{n}-S_{*}$ and $V_{n}=I_{n}-I_{*}$. We change the equilibrium $E_{3}(S^{*},I^{*})$ of model (9) and have the following result:

13 $$ \left \{ \textstyle\begin{array}{l} U_{n+1}=U_{n}+(h^{*}+h_{2})(-\mu_{2}U_{n}-\frac{\beta _{2}}{N_{2}}U_{n}V_{n}-\frac{\beta_{2}}{N_{2}}U_{n}I^{*}-\frac{\beta _{2}}{N_{2}}V_{n}S^{*}), \\ V_{n+1}=V_{n}+(h^{*}+h_{2})(\frac{\beta_{2}}{N_{2}}U_{n}V_{n}-(\gamma _{1}+\mu_{1})V_{n}+\frac{\beta_{2}}{N_{2}}U_{n}I^{*}+\frac{\beta _{2}}{N_{2}}V_{n}S^{*}), \end{array}\displaystyle \right . $$

which gives

$$\omega_{2}+P\bigl(h^{*}\bigr)\omega+Q\bigl(h^{*}\bigr)=0, $$

where

$$2+P\bigl(h^{*}\bigr)=\frac{\beta_{2}\mu_{2}(h_{2}+h^{*})}{\gamma_{2}\mu_{2}} $$

and

$$Q\bigl(h^{*}\bigr)=1-\frac{\beta_{2}\mu_{2}(h_{2}+h^{*})}{\gamma_{2}\mu _{2}}+\bigl(h_{2}+h^{*} \bigr)^{2}\bigl[\mu_{2}\beta_{2}- \mu_{2}(\mu_{2}+\gamma_{2})\bigr]. $$

It is easy to see that

$$\omega_{1,2}=\frac{-P(h^{*})\pm\sqrt{(P(h^{*}))^{2}-4Q(h^{*})}}{2}, $$

which gives

$$|\omega_{1,2}|=\sqrt{Q\bigl(h^{*}\bigr)},\quad\quad k=\frac{d|\omega _{1,2}|}{dh^{*}}\bigg|_{h^{*}=0}= \frac{\mu_{2}\beta_{2}}{2(\mu_{2}+\gamma_{2})}. $$

We remark that $(\mu_{2},N_{2},\beta_{2},h_{2},\gamma_{2})\in N^{+}$ and $\bigtriangleup<0$, and then we have

$$\frac{(\mu_{2}\beta_{2})^{2}}{(\gamma_{2}+\mu_{2})^{2}[\mu_{2}\beta _{2}-\mu_{2}(\mu_{2}+\gamma_{2})]}< 4. $$

Thus

$$P(0)=-2+\frac{(\mu_{2}\beta_{2})^{2}}{(\gamma_{2}+\mu_{2})^{2}[\mu _{2}\beta_{2}-\mu_{2}(\mu_{2}+\gamma_{2})]}\neq\pm2, $$

which means that

14 $$ \frac{\mu_{2}\beta_{2}}{(\gamma_{2}+\mu_{2})^{2}[\mu_{2}\beta _{2}-\mu_{2}(\mu_{2}+\gamma_{2})]}\neq\frac{j(\gamma_{2}+\mu _{2})}{\mu_{2}\beta_{2}},\quad j=2,3. $$

Hence, the eigenvalues $\omega_{1,2}$ of equilibrium $(0,0)$ of model (14) do not lie in the intersection when $h^{*}=0$ and (14) holds.

When $h^{*}=0$ we begin to study the model (14). Put

$$\begin{gathered} \alpha=\frac{(\mu_{2}\beta_{2})^{2}}{2(\gamma_{2}+\mu_{2})^{2}[\mu _{2}\beta_{2}-\mu_{2}(\mu_{2}+\gamma_{2})]}, \\ \beta= \frac{\mu_{2}\beta_{2} \sqrt{4 [\mu_{2}\beta_{2}-\mu_{2}(\mu _{2}+\gamma_{2})]-(\mu_{2}\beta_{2})^{2}}}{2(\gamma_{2}+\mu_{2})[\mu _{2}\beta_{2}-\mu_{2}(\mu_{2}+\gamma_{2})]}, \end{gathered} $$

and

$$T = \left ( \textstyle\begin{array}{c@{\quad}c} 0 & 1 \\ \beta& \alpha \end{array}\displaystyle \right ), $$

where *T* is invertible.

If we use the following transformation:

$$\begin{pmatrix}U_{n} \\ V_{n} \end{pmatrix} =T \begin{pmatrix}X_{n} \\ Y_{n} \end{pmatrix} , $$

then the model (14) gets the following form:

15 $$ \left \{ \textstyle\begin{array}{l} X_{n+1}=\alpha X_{n}-\beta Y_{n}+\bar{F}(X_{n},Y_{n}),\\ Y_{n+1}=\beta X_{n}+\alpha Y_{n}+\bar{G}(X_{n},Y_{n}), \end{array}\displaystyle \right . $$

where

$$\bar{F}(X_{n},Y_{n})=\frac{h_{2}\beta_{2}(1+\alpha)(\beta X_{n}Y_{n}+\alpha Y^{2}_{n})}{N_{2}\beta} $$

and

$$\bar{G}(X_{n},Y_{n})=\frac{-h_{2}\beta_{2}(\beta X_{n}Y_{n}+\alpha Y^{2}_{n})}{N_{2}}. $$

Moreover,

$$\begin{gathered} \bar{F}_{X_{n}X_{n}}=0,\qquad \bar{F}_{Y_{n}Y_{n}}= \frac{2h_{2}\beta _{2}\alpha(1+\alpha)}{N_{2}\beta_{2}}, \qquad\bar{F}_{X_{n}Y_{n}}=\frac {h_{2}\beta_{2}(1+\alpha)}{N_{2}}, \\ \bar{F}_{X_{n}X_{n}X_{n}}=\bar{F}_{X_{n}X_{n}Y_{n}}=\bar{F}_{X_{n}Y_{n}Y_{n}}=\bar{F}_{Y_{n}Y_{n}Y_{n}}=0, \\ \bar{G}_{X_{n}X_{n}}=0,\qquad \bar{G}_{Y_{n}Y_{n}}=- \frac{2h_{2}\beta _{2}\alpha}{N_{2}}, \qquad\bar{G}_{X_{n}Y_{n}}=-\frac{h_{2}\beta_{2}\beta}{N_{2}}, \\ \bar{G}_{X_{n}X_{n}X_{n}}=\bar{G}_{X_{n}X_{n}Y_{n}}=\bar{G}_{X_{n}Y_{n}Y_{n}}=\bar{G}_{Y_{n}Y_{n}Y_{n}}=0. \end{gathered} $$

Thus we have

$$a=-\operatorname{Re}\biggl[\frac{1-2\bar{\omega}}{1-\omega}\xi_{11}\xi_{20}\biggr]- \frac {1}{2}\|\xi_{11}\|^{2}-\| \xi_{02}\| ^{2}+\operatorname{Re}(\bar{\omega}\xi_{21}), $$

where

$$\begin{gathered} \xi_{02}=\frac{1}{8}\bigl[(\bar{F}_{X_{n}X_{n}}-\bar{F}_{Y_{n}Y_{n}}-2\bar{G}_{X_{n}Y_{n}})+(\bar{G}_{X_{n}X_{n}}-\bar{G}_{Y_{n}Y_{n}}+2\bar{F}_{X_{n}Y_{n}})i\bigr], \\ \xi_{11}=\frac{1}{4}\bigl[(\bar{F}_{X_{n}X_{n}}+\bar{F}_{Y_{n}Y_{n}})+(\bar{G}_{X_{n}X_{n}}+\bar{G}_{Y_{n}Y_{n}})i\bigr], \\ \xi_{20}=\frac{1}{8}\bigl[(\bar{F}_{X_{n}X_{n}}-\bar{F}_{Y_{n}Y_{n}}+2\bar{G}_{X_{n}Y_{n}})+(\bar{G}_{X_{n}X_{n}}-\bar{G}_{Y_{n}Y_{n}}-2\bar{F}_{X_{n}Y_{n}})i\bigr], \end{gathered} $$

and

$$\xi_{21}=\frac{1}{16}\bigl[(\bar{F}_{X_{n}X_{n}X_{n}}+\bar{F}_{X_{n}Y_{n}Y_{n}}+\bar{G}_{X_{n}X_{n}Y_{n}}+\bar{G}_{Y_{n}Y_{n}Y_{n}})\bigr]. $$

Therefore we have the following result.

### Theorem 4

*Let* $a \neq0$ *and* $h^{*}$ *change in a neighborhood of* $h_{***}$. *If the condition* (15) *holds*, *then model* (13) *undergoes a Hopf bifurcation at* $E_{2}(S^{*},I^{*})$. *If* $a>0$, *then the repelling invariant closed curve bifurcates from* $E_{2}$ *for* $h^{*}<0$. *If* $a<0$, *then an attracting invariant closed curve bifurcates from* $E_{2}$ *for* $h^{*}>0$.

## Conclusions

The paper investigated the basic dynamic characteristics of a Schrödinger-prey system with latent period. First, we applied the forward Euler scheme to a continuous-time SIR epidemic model and obtained the Schrödinger-prey system. Then the existence and local stability of the disease-free equilibrium and endemic equilibrium of the model are discussed. In addition, we chose *h* as the bifurcation parameter and studied the existence and stability of flip bifurcation and Hopf bifurcation of this model by using the center manifold theorem and the bifurcation theory. Numerical simulation results show that for the model (2) there occurs a flip bifurcation and a Hopf bifurcation when the bifurcation parameter *h* passes through the respective critical values, and the direction and stability of flip bifurcation and Hopf bifurcation can be determined by the sign of $\alpha_{2}$ and *a*, respectively. Apparently there are more interesting problems as regards this Schrödinger-prey system with latent period which deserve further investigation.
